# Supplementary material for: Do ecological characteristics drive the prevalence of Panulirus argus virus 1 (PaV1) in juvenile Caribbean spiny lobsters in a tropical reef lagoon?
Source: PLoS One. 2020 Feb 28;15(2):e0229827. doi: 10.1371/journal.pone.0229827 (PMC7048287; doi:10.1371/journal.pone.0229827)
Supplement: S1 Fig — Habitat assessment score in the three sampling zones (zone A: red columns; zone B: blue columns; zone C: gray columns) in four sampling periods: June 2016 (J’16), November 2016 (N’16), June 2017 (J’17) and November 2017 (N’17). Error bars denote 95% confidence intervals. (PDF) [file pone.0229827.s002.pdf]

S1 Fig.

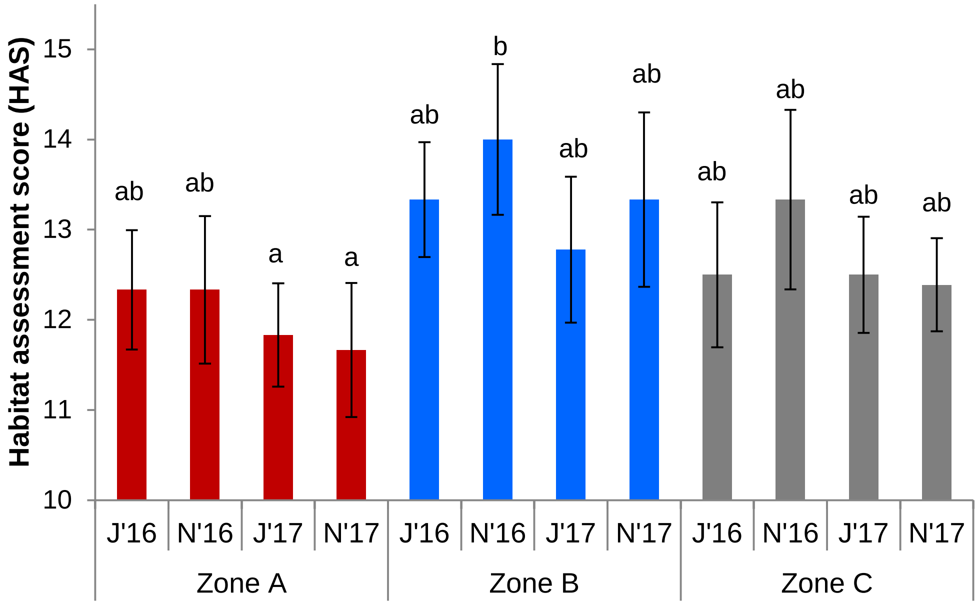

S1 Figure. Temporal and spatial variation in habitat complexity.

Habitat assessment score of the three sampling zones (zone A: red columns; zone B: blue columns; zone C: gray columns) in the Puerto Morelos reef lagoon in four sampling periods: June 2016 (J'16), November 2016 (N'16), June 2017 (J'17) and November 2017 (N'17). Error bars denote 95% confidence intervals. Different letters above bars denote significant differences.
