# Supplementary material for: Do ecological characteristics drive the prevalence of Panulirus argus virus 1 (PaV1) in juvenile Caribbean spiny lobsters in a tropical reef lagoon?
Source: PLoS One. 2020 Feb 28;15(2):e0229827. doi: 10.1371/journal.pone.0229827 (PMC7048287; doi:10.1371/journal.pone.0229827)
Supplement: S3 Fig — Percentage of lobsters showing clinical signs of PaV1 infection in three sampling zones (zone A: red columns; zone B: blue columns; zone C: gray columns) in the Puerto Morelos reef lagoon, in seven sampling periods: June 2016 (J’16), November 2016 (N’16), June 2017 (J’17), November 2017 (N’17), November 2018 (N’18), June 2019 (J’19), and November 2019 (N’19). Numbers in parentheses below dates are sample sizes. Error bars denote 95% confidence intervals. (PDF) [file pone.0229827.s004.pdf]

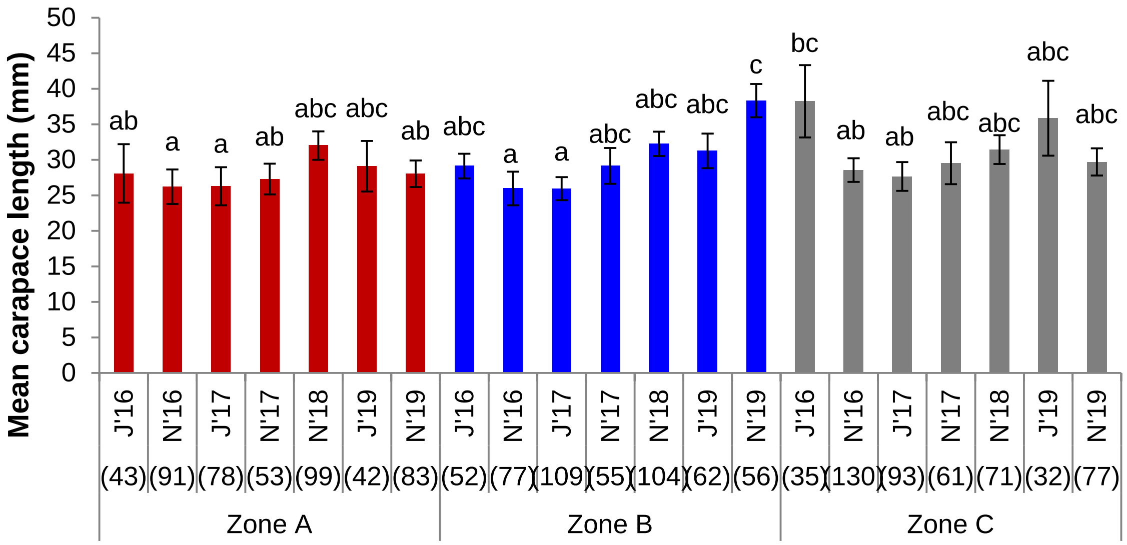

S3 Fig. Lobster mean size by sampling zone and period.

Mean size (carapace length, mm) of lobsters sampled in three sampling zones (zone A: red columns; zone B: blue columns; zone C: gray columns) in the Puerto Morelos reef lagoon, in seven sampling periods: June 2016 (J'16), November 2016 (N'16), June 2017 (J'17), November 2017 (N'17), November 2018 (N'18), June 2019 (J'19), and November 2019 (N'19). Numbers in parentheses below dates are sample sizes. Error bars denote 95% confidence intervals. Different letters above bars denote significant differences.
