# Supplementary material for: Do ecological characteristics drive the prevalence of Panulirus argus virus 1 (PaV1) in juvenile Caribbean spiny lobsters in a tropical reef lagoon?
Source: PLoS One. 2020 Feb 28;15(2):e0229827. doi: 10.1371/journal.pone.0229827 (PMC7048287; doi:10.1371/journal.pone.0229827)
Supplement: S1 Table — Invertebrate species (in alphabetical order within higher taxa) and number of individuals observed by sampling zone (zones A, B, and C) across four sampling periods (June and November of 2016 and 2017), Puerto Morelos reef lagoon (N = 18 quadrats, 2 m × 2 m, per zone per period). (PDF) [file pone.0229827.s005.pdf]

S1 Table. Invertebrate species list.

Invertebrate species (in alphabetical order within higher taxa) and number of individuals observed by sampling zone (zones A, B, and C) across four sampling periods (June and November of 2016 and 2017), Puerto Morelos reef lagoon (N = 18 quadrats, 2 m × 2 m, per zone per period)

Species	Zone A	Zone B	Zone C	Total
CNIDARIA: ANTHOZOA				
Actinostella flosculifera (Le Sueur, 1817)	31	0	33	64
Anemonia sargassensis Hargitt, 1908	1	1	0	2
Bartholomea annulata (Le Sueur, 1817)	12	0	3	15
Condylactis gigantea (Weinland, 1860)	4	2	0	6
Dichocoenia stokesii (Milne Edwards & Haime, 1848)	6	0	0	6
Isarachnanthus nocturnus (Hartog, 1977)	0	1	1	2
Ricordea florida Duchassaing & Michelotti, 1860	0	0	1	1
Unidentified anemone	0	2	2	4
ANNELIDA: POLYCHAETA				
Eupolymnia crassicornis (Schmarda, 1861)	12	39	19	70
Hermodice carunculata (Pallas, 1766)	11	7	7	25
Loimia medusa (Savigny, 1822)	0	0	8	8
Pomatostegus stellatus (Abildgaard, 1789)	0	0	2	2
Spirobranchus giganteus (Pallas, 1766)	0	1	0	0
CRUSTACEA: DECAPODA				
Alpheus armatus Rathbun, 1901	1	0	1	2
Calcinus tibicen (Herbst, 1791)	37	21	14	72
Clibanarius tricolor (Gibbes, 1850)	144	143	149	436
Epialtus sp.	0	0	5	5
Macrocoeloma trispinosum (Latreille, 1825)	2	0	3	5
Mithraculus coryphe (Herbst, 1801)	4	7	0	11
Mithraculus forceps A. Milne-Edwards, 1875	7	7	1	15
Mithraculus sculptus (Lamarck, 1818)	64	40	59	163
Mithrax sp.	0	0	4	4
Omalacantha bicornuta (Latreille, 1825)	2	0	0	2
Paguristes anomalus Bouvier, 1918	0	10	4	14
Paguristes puncticeps Benedict, 1901	35	57	47	139
Paguristes sp.	0	0	3	3
Pagurus annulipes (Stimpson, 1860)	81	81	115	277
Pagurus brevidactylus (Stimpson, 1859)	263	257	237	757
Pagurus sp.	0	2	0	3
Panulirus argus (Latreille, 1804)*	1	0	0	1
Phimochirus holthuisi (Provenzano, 1961)	2	0	0	2
Pitho lherminieri (Desbonne in Desbonne & Schramm, 1867)	27	100	23	150
Pitho mirabilis (Herbst, 1794)	0	31	6	37
Portunus sp.	0	0	1	1

Scyllarides aequinoctialis (Lund, 1793)	0	0	1	1
Stenopus hispidus (Olivier, 1811)	1	0	0	1
Stenorhynchus seticornis (Herbst, 1788)	0	0	1	1
Teleophrys ruber (Stimpson, 1871)	2	0	1	3
Unidentified Diogenid	0	0	3	3
Unidentified Grapsid	0	0	1	1
Unidentified Xanthoid 1	1	0	2	3
Unidentified Xanthoid 2	1	0	0	1
Unidentified Xanthoid 3	0	0	4	4
CRUSTACEA: STOMATOPODA				
Neogonodactylus curacaoensis (Schmitt, 1924)	0	2	0	2
Neogonodactylus oerstedii (Hansen, 1895)	2	3	7	12
Pseudosquilla ciliata (Fabricius, 1787)	1	4	0	5
Unidentified Stomatopod	0	0	7	7
ECHINODERMA: ASTEROIDEA				
Oreaster reticulatus (Linnaeus, 1758)	0	2	3	5
ECHINODERMA: ECHINOIDEA				
Clypeaster subdepressus (Gray, 1825)	1	0	0	1
Echinometra viridis A. Agassiz, 1863	1	0	0	1
Eucidaris tribuloides (Lamarck, 1816)	1	0	2	3
Lytechinus variegatus (Lamarck, 1816)	34	23	0	57
Meoma ventricosa (Lamarck, 1816)	0	0	1	1
Tripneustes ventricosus (Lamarck, 1816)	2	1	0	3
ECHINODERMA: HOLOTHUROIDEA				
Holothuria sp.	0	1	0	1
ECHINODERMA: OPHIUROIDEA				
Ophiocoma echinata (Lamarck, 1816)	1	11	0	12
Ophiocoma sp. 1	0	1	1	2
Ophiocoma sp. 2	6	0	0	6
Ophioderma appressa (Say, 1825)	41	224	18	283
Ophioderma rubicunda Lütken, 1856	0	37	0	37
Ophioderma sp.	44	180	23	247
Ophionereis sp.	2	0	0	2
Ophiothrix (Acanthophiothrix) suensoni Lütken, 1856	2	0	2	4
Unidentified Ophiurid 1	0	0	1	1
Unidentified Ophiurid 2	0	3	0	3
Unidentified Ophiurid 3	0	0	1	1
MOLLUSCA: BIVALVIA				
Arca zebra Swainson, 1833	6	21	5	32
Caribachlamys pellucens (Linnaeus, 1758)	0	0	1	1
Pinna carnea Gmelin, 1791	1	7	2	10
Unidentified Bivalve	2	15	1	18
MOLLUSCA: GASTROPODA				
Calliostoma sp.	4	0	0	4
Cerithium litteratum (Born, 1778)	162	121	103	386

Cerithium sp.	32	40	48	120
Cittarium pica (Linnaeus, 1758)	18	55	33	106
Conus sp.	0	1	0	1
Cypraea sp.	0	1	0	1
Diodora cayenensis (Lamarck, 1822)	9	60	8	77
Diodora sp.	42	11	2	55
Fissurella sp.	3	2	4	9
Jujubinus sp.	7	0	0	7
Lithopoma phoebium (Röding, 1798)	41	81	39	161
Lithopoma tectum (Lightfoot, 1786)	10	34	16	60
Littorina sp.	6	1	9	16
Lobatus gigas (Linneaus, 1758)	0	0	3	3
Lobatus raninus (Gmelin, 1791)	0	1	0	1
Modulus modulus (Linnaeus, 1758)	53	60	166	279
Prunum pruinosum (Hinds, 1844)	4	2	1	7
Smaragdia viridis (Linnaeus, 1758)	129	132	126	387
Strombus sp.	2	3	0	5
Tegula fasciata (Born, 1778)	317	397	333	1047
Vasum muricatum (Born, 1778)	2	0	1	3
Unidentified Gastropod 1	0	6	1	7
Unidentified Gastropod 2	1	0	1	2
Unidentified Gastropod 3	3	0	0	3
Unidentified Lepetellid	0	0	17	17
Unidentified Nudibranch	0	0	1	1
**Total individuals**	1744	2353	1749	5846
**Total species**	60	53	67	96

*very small juvenile
