# Supplementary material for: Do ecological characteristics drive the prevalence of Panulirus argus virus 1 (PaV1) in juvenile Caribbean spiny lobsters in a tropical reef lagoon?
Source: PLoS One. 2020 Feb 28;15(2):e0229827. doi: 10.1371/journal.pone.0229827 (PMC7048287; doi:10.1371/journal.pone.0229827)
Supplement: Attachment — Submitted filename: Davies et al comments.pdf [file pone.0229827.s006.pdf]

Manuscript

Click here to download Manuscript Manuscript.docx

Δ change this title. It was not really a monitoring study per se.

Monitoring the role of biodiversity and small-scale habitat change in disease prevalence among juvenile Caribbean spiny lobsters

this implies the habitat changed. I would change this word to 'differences'

Short title: Biodiversity and habitat features associated with disease in juvenile Caribbean spiny lobsters

Charlotte E. Davies[1,2], Patricia Briones-Fourzán[1*], Cecilia Barradas-Ortiz[1], Fernando Negrete-Soto[1], Gema Moo-Cocom[1], and Enrique Lozano-Álvarez[1]

[1] Unidad Académica de Sistemas Arrecifales, Instituto de Ciencias del Mar y Limnología, Universidad Nacional Autónoma de México, Puerto Morelos, Quintana Roo, 77580 Mexico

[2] *Current address*: Department of Biosciences, College of Science, Swansea University, Singleton Park, Swansea, Wales UK, SA2 8PP

* Corresponding author

E-mail: briones@cmarl.unam.mx (PB-F)

Abstract

PaV1 is a pathogenic virus found only to infect Caribbean spiny lobsters *Panulirus argus*, a major fishing resource and important benthic mesopredator. PaV1 prevalence is higher in smaller, juvenile lobsters and in more vegetated habitats, suggesting that vegetation may be acting as an environmental reservoir for the disease. *Panulirus argus* has a complex life history with several ontogenetic habitat changes (from oceanic waters to shallow, vegetated habitats, to crevice-type shelters, to coral reef habitats) that may have implications for disease dynamics, which may be further affected by species diversity in a local community. To increase insight into PaV1 within *P. argus* populations in the Caribbean, we targeted a population of juvenile *P. argus* in a shallow reef lagoon. Since juveniles have more limited home ranges than adults, we expected that local levels of prevalence of PaV1 would be influenced by habitat characteristics and/or species diversity of surrounding communities. Therefore, we assessed habitat complexity, cover of different substrates, and invertebrate community composition in three sampling zones of the reef lagoon across four periods (June and November of 2016 and 2017). Lobsters were sampled concurrently with these assessments plus two additional periods (November 2018 and June 2019) to estimate clinical prevalence of PaV1. Consistent with previous studies, probability of clinical infection decreased with increasing lobster size. However, zone had no effect on probability of infection, i.e., variations in habitat characteristics did not appear to be sufficiently large so as to influence prevalence of PaV1. This result possibly reflects the dominance of marine vegetation (seagrass and macroalgae) in all three zones at all sampling periods (>73% cover). Although sampling period affected the probability of infection with PaV1, clinical prevalence was higher in only one of our six sampling periods (November 2017), suggesting that the infection is settled down to an enzootic level.

KEY WORDS: disease ecology, marine protected area, spiny lobster, habitat assessment,

parasites, invertebrates, biodiversity, reef ecology, seasonal variation, PaV1

Introduction

The Caribbean spiny lobster *Panulirus argus* (Latreille, 1804) is an important fishing resource throughout the Wider Caribbean region [1]. This species has a complex life history with several ontogenetic habitat changes. After an extended larval period that develops in oceanic waters for 5 to 7 months, the postlarvae of *P. argus* return to the coast and settle in shallow, vegetated habitats (seagrass meadows, macroalgal beds), where the small juveniles (6 to ~20 mm carapace length, CL) dwell for a few months. Upon outgrowing the protection afforded by the vegetation, juvenile lobsters seek structured crevice-type shelters within or adjacent to the seagrass meadows. Further on, the subadults (~50 to 80 mm CL) start migrating to the coral reefs, which the adults (>80 mm CL) inhabit [2].

Panulirus argus are omnivorous mesopredators and play an important ecological role in Caribbean coral reefs systems [3–5], but they are also susceptible to parasites and diseases. For example, they are hosts to *Panulirus argus* virus 1 (PaV1), the first known naturally occurring virus of a lobster [6], and are second intermediate hosts to *Cymatocarpus solearis*, a digenean trematode that infects sea turtles [7–9]. PaV1 was first discovered in Florida (USA) in 2000 [10] and shortly thereafter in Puerto Morelos (México) in 2001 [11]. The main clinical/gross sign of infection is a 'milky' white hemolymph, immediately visible through the translucent membrane between the carapace and abdomen [12]. PaV1 has only been found to affect *P. argus* but is currently widespread throughout the Caribbean [13,14], linked to various factors. Principally, PaV1 prevalence has been correlated with lobster size, being found more in smaller, juvenile lobsters [15,16], and with habitat, being found more in highly vegetated habitats [9,17,18],

suggesting that vegetation may be acting as an environmental reservoir for the disease. In contrast, *C. solearis* appears to be more prevalent in adult lobsters than in juveniles [8] irrespective of habitat type [9].

Although Caribbean spiny lobsters are gregarious, healthy lobsters tend to avoid heavily PaV1-infected conspecifics, which may help curb prevalence levels [19–22]. However, infections involve interactions not only between the parasite and the host, but among networks of species [23]. For example, the life cycle of *C. solearis* involves three successive hosts. Many digeneans in the suborder Xiphidiata, to which *C. solearis* belongs, can use more than one species of gastropods as first intermediate hosts [24], but the first intermediate hosts of *C. solearis* remain unknown. After emerging from the first intermediate hosts, Xiphidiate cercariae penetrate the bodies of the second intermediate hosts, which for *C. solearis* include *P. argus* and several other decapod crustaceans [7,25,26]. This is because cercariae are more specific to the host's environment than to the host itself [27]. Therefore, gastropods that co-occur with spiny lobsters are more likely to be first intermediate hosts of *C. solearis* than gastropods that use other habitats [9].

which? citation?

this connection is weak. Viruses are typically directly transmitted so this is irrelevant. Omit

Even in host-specific disease systems (such as PaV1/*P. argus*), species diversity of surrounding communities may affect disease dynamics in different ways [23,28,29]. For example, non-host species may reduce the probability of encounter between hosts, and if non-host species are prey or mutualists of hosts, they can reduce host stress, potentially increasing the efficacy of the host immune response [23]. Additionally, some non-host species may act as reservoirs of pathogens [30], although reservoirs of PaV1 and effects upon the ecosystem as a whole are relatively unknown. As previously noted, some studies had noted a higher PaV1 prevalence in densely-vegetated areas compared to poorly-vegetated areas, suggesting that vegetation may be acting as an environmental reservoir for the disease [17,18]. This notion has

need examples and further explanation of how these would work.

lines 86-92. Needs to be revised for clarity & brevity.

been supported by a recent study [9] in which the probability of infection with PaV1 was also found to be higher in lobsters inhabiting more vegetated habitats, but further proposing that either marine vegetation or fauna that live associated with vegetated habitats, or both, may be reservoirs of PaV1.

The current study aims to discover more about PaV1 and *C. solearis* within populations of *P. argus* in the Caribbean, by targeting a population of juvenile lobsters in a shallow reef lagoon. We expected to find low prevalence levels of *C. solearis* but high prevalence levels of PaV1 in this reef lagoon relative to habitats used by adult lobsters [9,17,18]. Since juveniles of *P. argus* have more limited home ranges than adults do [31,32], we expected local levels of prevalence of PaV1 to be influenced by small scale habitat characteristics (e.g. the presence and relative abundance of marine vegetation) and/or species diversity of local communities [9]. However, whilst the roles of habitat and local communities may be crucial in understanding disease ecology, the role of temporal variation is equally as important, and often overlooked in short-term or snapshot studies. Therefore, we also aimed to decipher temporal variation in the disease prevalence in this reef lagoon.

Materials and Methods

Study area

The study was carried out in the Puerto Morelos Reef National Park, located at the northernmost section of the Mesoamerican Barrier Reef, on the Mexican Caribbean coast. This marine system (centered at 20°52'N 86°5'W) consists of an interrupted fringing reef located at a distance of ~0.5 to 2 km from the coast [33] (Fig 1). The reef reduces wave energy, allowing the presence of a shallow reef lagoon (~5 m in maximum depth) where seagrass meadows dominated
