# Supplementary material for: Do ecological characteristics drive the prevalence of Panulirus argus virus 1 (PaV1) in juvenile Caribbean spiny lobsters in a tropical reef lagoon?
Source: PLoS One. 2020 Feb 28;15(2):e0229827. doi: 10.1371/journal.pone.0229827 (PMC7048287; doi:10.1371/journal.pone.0229827)
Supplement: Attachment — Submitted filename: PLoS editorial comment_Disease prevalence in spiny lobsters.docx [file pone.0229827.s007.docx]

**PLoS editorial comment_Disease prevalence in spiny lobsters**

Overall, a well written and interesting manuscript. The manuscript lacks an ethics statement. See the online submission guidelines for more information on what is required, but there should be a short sub-section, or just a few sentences, in the methods section on all the permits and permissions needed to undertake the research, including what department or authority issued them and associated permit numbers.

Line 22: Just use the abbreviation P. argus here and need a comma after ‘life history’

Line 27: Would be good to say where the study took place (Mexico)

Line 32: Need a comma after ‘assessments’ and after the dates

Lines 39-40: I find this last bit a little awkward ‘settled down’….is there another way to phrase this?

Line 46: delete ‘wider’

Line 52: replace ‘Further on’ with ‘Later’ and delete the ‘the’ before ‘coral reefs’

Lines 54, 56: Again, just use the abbreviated scientific name now that the full name has already been given.

Line 66: Need a comma at the end of the line

Line 86: Write as ‘reported a higher’

Line 95: Need a comma before ‘but’

Line 119: Write as ‘lagoon lacks structured’

Line 123: Write as ‘then on the PaV1’ or something like that

Line 127: ‘depth, density and height’

Line 130: semicolon before and zone C

Line 141: ‘for two contrasting’

Line 143: ‘in each zone’

Line 145: move this bit ‘was estimated….’ Up to after ‘substrate’ in line 144

Line 165: ‘prio to sampling’

Line 168: delete ‘both’

Line 171: ‘scuba’ is no longer considered an acronym, but just a regular word so it does not have to be in all capitals; ‘during the same four’

Line 176: the abbreviation CL was already established on line 50 so it does not need to be defined here. Additional info on what CL actually means should be moved to line 50.

Line 179: Delete of’ before `1mm

Line 184: Need to spell out what PCR stands for

Line 198: This should be spelled out on line 184; just use PCR here

Line 216: ‘type of substrate

Line 240: ‘were log-transformed’

Line 243: ‘In the first model’

Line 260: GLM has already been established, so you can just use that here. If you want to spell it out, then delete GLM; you don’t need both.

Line 273: add ‘,respectively’ at the end of the sentence

Line 297: HAS has already been established, so you can just use that here. If you want to spell it out, then delete HAS; you don’t need both

Line 324: GLM has already been established, so you can just use that here. If you want to spell it out, then delete GLM; you don’t need both.

Line 349: Abbreviate the scientific name

Line 390, 391: Do not capitalise ‘zone’

Line 391: Abbreviate the scientific name

Line 397: Abbreviate the scientific name

Line 411: ‘type of substrate’

Line 412: ‘for four different’; Delete ‘on the same zone’ as this does not make sense.

Line 413: Start the next sentence ‘Lobsters were sampled…..plus two additional periods in each zone’

Line 418: ‘variation in habitat’

Line 432: A new paragraph might be needed here….up to the authors.

Line 442: ‘were clearly related’

Line 456: delete ‘the proximity of’

Line 458: write as ‘with a diseased lobster (e.g. due to the risk of predation)

Line 460: Should this virus name be capitalised?

Line 467: Full stop after the stone crab name, start the next sentence ‘However……’

Line 468: Delete the ‘However’ here and just say ‘None of these…’

Line 471: Need to start a new paragraph here ‘In conjunction’

Line 475: Need a comma after ‘period’

Lines 477-478: Need to abbreviate these scientific names as they have already been given in full on lines 316-319

Line 480: Write as ‘techniques, such as endpoint PCR……’

Line 489: As noted for the abstract, I find this phrase awkward. But if the authors want to keep it it should be ‘has settled down’

Line 498: write as ‘it provides information on how widespread’

Line 507: ‘for the biological’

Line 514: Should be ‘new normal’
