# Supplementary material for: Do ecological characteristics drive the prevalence of Panulirus argus virus 1 (PaV1) in juvenile Caribbean spiny lobsters in a tropical reef lagoon?
Source: PLoS One. 2020 Feb 28;15(2):e0229827. doi: 10.1371/journal.pone.0229827 (PMC7048287; doi:10.1371/journal.pone.0229827)
Supplement: Attachment — Submitted filename: Response to Reviewers.docx [file pone.0229827.s008.docx]

From:	"PLOS ONE" [<em@editorialmanager.com>](mailto:em@editorialmanager.com)

Subject: PLOS ONE Decision: Revision required [PONE-D-19-25184] - [EMID:78acbfad5d395c28] Date:	Wed, October 23, 2019 12:26 am

To:	Patricia Briones-Fourzán [<briones@cmarl.unam.mx>](mailto:briones@cmarl.unam.mx)

PONE-D-19-25184

Monitoring the role of biodiversity and small-scale habitat change in disease prevalence among juvenile

Caribbean spiny lobsters

PLOS ONE

Dear Dr. Briones-Fourzán,

Thank you for submitting your manuscript to PLOS ONE. After careful consideration, we feel that it has merit but does not fully meet PLOS ONE’s publication criteria as it currently stands. Therefore, we invite you to submit a revised version of the manuscript that addresses the points raised during the review process.

I found this to be an interesting and well written study. However, both reviewers have identified substantial issues with the manuscript that need to be rectified. The main issue with the study is that it seems the two sites were too homogenous to actually create any differences. Both reviewers have provided detailed comments to assist the authors in their revision and I have provided editorial comments. The authors should consider all the comments provided.

We would appreciate receiving your revised manuscript by Dec 07 2019 11:59PM. When you are ready to submit your revision, log on to [https://www.editorialmanager.com/pone/](http://www.editorialmanager.com/pone/) and select the 'Submissions Needing Revision' folder to locate your manuscript file.

If you would like to make changes to your financial disclosure, please include your updated statement in your cover letter.

To enhance the reproducibility of your results, we recommend that if applicable you deposit your laboratory protocols in protocols.io, where a protocol can be assigned its own identifier (DOI) such that it can be cited [independently in the future. For instructions see: http://journals.plos.org/plosone/s/submission-guidelines#loc- laboratory-protocols](http://journals.plos.org/plosone/s/submission-guidelines#loc-laboratory-protocols)

Please include the following items when submitting your revised manuscript:

A rebuttal letter that responds to each point raised by the academic editor and reviewer(s). This letter should be uploaded as separate file and labeled 'Response to Reviewers'.

A marked-up copy of your manuscript that highlights changes made to the original version. This file should be uploaded as separate file and labeled 'Revised Manuscript with Track Changes'.

An unmarked version of your revised paper without tracked changes. This file should be uploaded as separate file and labeled 'Manuscript'.

Please note while forming your response, if your article is accepted, you may have the opportunity to make the peer review history publicly available. The record will include editor decision letters (with reviews) and your responses to reviewer comments. If eligible, we will contact you to opt in or out.

We look forward to receiving your revised manuscript. Kind regards,

Heather M. Patterson, Ph.D.

Academic Editor

PLOS ONE

**Journal Requirements:**

**Our responses to comments are in green Calibri font. Please note that we numbered the reviewers’ comments to facilitate cross-referencing them. In our Responses, References which appear in the revised manuscript are followed by the corresponding number (e.g. [16]). References which do not appear in the References section of the revised manuscript appear at the end of this section of Responses.**

1. When submitting your revision, we need you to address these additional requirements.

Please ensure that your manuscript meets PLOS ONE's style requirements, including those for file naming. The PLOS ONE style templates can be found at

http://www.journals.plos.org/plosone/s/file?id=wjVg/PLOSOne_formatting_sample_main_body.pdf and http://www.journals.plos.org/plosone/s/file?id=ba62/PLOSOne_formatting_sample_title_authors_affiliations.pdf

**R. PLoS One’s style requirements have been now carefully followed. Also, according to PACE, all figures meet PLOS requirements**

2. In your Methods section, please provide additional information regarding the permits you obtained for the work. Please ensure you have included the full name of the authority that approved the field site access and, if no permits were required, a brief statement explaining why.

**R. Permit information has now been added, both in Methods and in Ethics Statement.**

3. We note that Figure(s) 1 in your submission contain [map/satellite] images which may be copyrighted. All PLOS content is published under the Creative Commons Attribution License (CC BY 4.0), which means that the manuscript, images, and Supporting Information files will be freely available online, and any third party is permitted to access, download, copy, distribute, and use these materials in any way, even commercially, with proper attribution. For these reasons, we cannot publish previously copyrighted maps or satellite images created using proprietary data, such as Google software (Google Maps, Street View, and Earth). For more information, see our copyright guidelines: [http://journals.plos.org/plosone/s/licenses-and-copyright.](http://journals.plos.org/plosone/s/licenses-and-copyright)

We require you to either (1) present written permission from the copyright holder to publish these figures specifically under the CC BY 4.0 license, or (2) remove the figures from your submission:

a) You may seek permission from the original copyright holder of Figure(s) [#] to publish the content specifically under the CC BY 4.0 license.

We recommend that you contact the original copyright holder with the Content Permission Form

(http://journals.plos.org/plosone/s/file?id=7c09/content-permission-form.pdf) and the following text:

“I request permission for the open-access journal PLOS ONE to publish XXX under the Creative Commons Attribution License (CCAL) CC BY 4.0 [(http://creativecommons.org/licenses/by/4.0/).](http://creativecommons.org/licenses/by/4.0/)) Please be aware that this license allows unrestricted use and distribution, even commercially, by third parties. Please reply and provide explicit written permission to publish XXX under a CC BY license and complete the attached form.”

Please upload the completed Content Permission Form or other proof of granted permissions as an "Other" file with your submission.

In the figure caption of the copyrighted figure, please include the following text: “Reprinted from [ref] under a

CC BY license, with permission from [name of publisher], original copyright [original copyright year].”

b) If you are unable to obtain permission from the original copyright holder to publish these figures under the CC BY 4.0 license or if the copyright holder’s requirements are incompatible with the CC BY 4.0 license, please either i) remove the figure or ii) supply a replacement figure that complies with the CC BY 4.0 license. Please check copyright information on all replacement figures and update the figure caption with source information. If applicable, please specify in the figure caption text when a figure is similar but not identical to the original image and is therefore for illustrative purposes only.

The following resources for replacing copyrighted map figures may be helpful:

USGS National Map Viewer (public domain): <http://viewer.nationalmap.gov/viewer/>

The Gateway to Astronaut Photography of Earth (public domain): <http://eol.jsc.nasa.gov/sseop/clickmap/>

Maps at the CIA (public domain): [https://www.cia.gov/library/publications/the-world-factbook/index.html](http://www.cia.gov/library/publications/the-world-factbook/index.html) and

[https://www.cia.gov/library/publications/cia-maps-publications/index.html](http://www.cia.gov/library/publications/cia-maps-publications/index.html) NASA Earth Observatory (public domain): <http://earthobservatory.nasa.gov/> Landsat: <http://landsat.visibleearth.nasa.gov/>

USGS EROS (Earth Resources Observatory and Science (EROS) Center) (public domain): [http://eros.usgs.gov/#](http://eros.usgs.gov/) Natural Earth (public domain): <http://www.naturalearthdata.com/>

**R. Figure 1 is not reprinted from a copyrighted source; it was produced using original information generated in my own Institute by Servicio Académico de Monitoreo Meteorológico y Oceanográfico (SAMMO) (Academic Service of Meteorologic and Oceanographic Monitoring). This source has been added to figure caption. A content permission form signed by the officer in charge of SAMMO is being uploaded with the revised ms.**

**Comments to the Author**

1. Is the manuscript technically sound, and do the data support the conclusions?

The manuscript must describe a technically sound piece of scientific research with data that supports the conclusions. Experiments must have been conducted rigorously, with appropriate controls, replication, and sample sizes. The conclusions must be drawn appropriately based on the data presented.

Reviewer #1: No

Reviewer #2: Yes

2. Has the statistical analysis been performed appropriately and rigorously? Reviewer #1: Yes

Reviewer #2: Yes

3. Have the authors made all data underlying the findings in their manuscript fully available?

The [PLOS Data policy](http://www.plosone.org/static/policies.action#sharing) requires authors to make all data underlying the findings described in their manuscript fully available without restriction, with rare exception (please refer to the Data Availability Statement in the manuscript PDF file). The data should be provided as part of the manuscript or its supporting information, or deposited to a public repository. For example, in addition to summary statistics, the data points behind means, medians and variance measures should be available. If there are restrictions on publicly sharing data—e.g. participant privacy or use of data from a third party—those must be specified.

Reviewer #1: Yes

Reviewer #2: Yes

4. Is the manuscript presented in an intelligible fashion and written in standard English?

PLOS ONE does not copyedit accepted manuscripts, so the language in submitted articles must be clear, correct, and unambiguous. Any typographical or grammatical errors should be corrected at revision, so please note any specific errors here.

Reviewer #1: Yes

Reviewer #2: Yes

5. Review Comments to the Author

Please use the space provided to explain your answers to the questions above. You may also include additional comments for the author, including concerns about dual publication, research ethics, or publication ethics. (Please upload your review as an attachment if it exceeds 20,000 characters)

**Reviewer #1: Overview:** The manuscript provides an analysis of the habitat types, locations, and temporal components on the prevalence of PaV1 in spiny lobsters, Panulirus argus, from Puerto Morales, Mexico. It also attempts to associate possible differences in biodiversity between locations and prevalence. Although this was a large undertaking, it does present a few significant design flaws that detract from the findings. The locations selected for sampling were essentially too homogenous and so no differences were found among locations and few differences in temporal data. In effect, the locations A, B, and C are subsamples within the narrow confines of the reef-flat seagrass complex and have few differences. For example, seagrass was >73% cover in all transects and zones. That alone accounts for the lack of relationships found in the variables and their potential relationship with PaV1. Moreover, the postlarvae settle in seagrass and the early benthic juveniles and larger juveniles stay in this nursery habitat, leaving it as subadults or adults. Because the virus has a predilection for juveniles and juveniles prefer seagrass, the virus will be found in juveniles in seagrass. Thus relating the virus to seagrass habitat is a tautology, and thus not very interesting. This conclusion is addressed somewhat in the Discussion, but it would be better to be up front with this in the M&M, Results, and Discussion.

**R. We respectfully disagree that relating the virus to seagrass habitat is a tautology. Please see more extended response to Comment # 8 below.**

Comments to the Author

1. Is the ms technically sound and do the data support the conclusions?

1. The ms has a significant design flaw in that the locations selected for sampling were essentially subsamples. There was little heterogeneity in the habitat.

**R. We respectfully disagree. The locations selected for sampling were not essentially subsamples. Please see the more extended response to comment # 17 below.**

5. Review comments to the author

2. The title does not reflect the nature of the work that was done. The short title is better and I would place the habitat features first in the title. Monitoring wasn’t really the objective here and it’s overstated in the title. Another title could be “An investigation into the ecological determinants in the prevalence of PaV1 in juvenile lobsters from Puerto Morales, Mexico.”

**R. The title has been changed to: “Ecological determinants in the prevalence of PaV1 in juvenile Caribbean spiny lobsters in a tropical reef lagoon.”**

3. The work on Cymatocarpus solearis does not contribute to the overall paper. I suggest dropping it because it’s superfluous. There weren’t enough infected lobsters to gain any additional understanding of this parasite in the lobster intermediate host.

**R. Agree. The work on *C. solearis* has been removed from the manuscript.**

4. Line 39: “infection” is misstated here. I would use the term “virus” or “pathogen” rather than infection. See also Line 489. The prevalence of the virus has fluctuated but is now established?

**R. “infection” has been changed to “pathogen” and the sentence was rephrased to “suggesting that the pathogen has leveled off to an enzootic level”.**

5. Biodiversity is mentioned in the title, but the effect of biodiversity is not mentioned in the abstract.

**R. The title has been changed to: “Ecological determinants in the prevalence of PaV1 in juvenile Caribbean spiny lobsters in a tropical reef lagoon.”**

6. Line 61: garbled sentence. PaV1 only infects P. argus and is currently widespread…

**R. Sentence has been changed as suggested.**

7. Line 65 and in the Discussion: “vegetation may be acting as an environmental reservoir for the disease.” There are a few issues with this statement. First, the vegetation is likely not the reservoir, the reservoir is other infected lobsters shedding virus in this habitat. This could be tested by testing eDNA samples for the presence of the virus in the vegetated habitat as opposed to infected lobsters. This was not done. Second, disease is what happens in individual lobsters, the pathogen or agent is what is being sheltered or transmitted by a reservoir. Finally, lobsters with active, end-stage infections do not move much (morbidity) and thus wouldn’t be expected to move out of this habitat. The above points may be relevant to restructuring the Discussion.

**R. Marine vegetation can certainly be an environmental reservoir for bacteria and viruses (see Small and Pagenkopp 2013 [24]; Sweet et al. 2013). Viruses can adsorb to plant surfaces (Gerba 1984) [61] and shading can protect virions from UV radiation (e.g. Raymond et al. 2005). Indeed, eDNA could help test this hypothesis, but that was not an objective of this study.**

8. Lines 89-92: again, the issue with the tautology.

**R. We respectfully disagree that relating the virus to seagrass habitat is a tautology. It is true that the virus has a predilection for juveniles and that juveniles prefer seagrass; however, previous studies (Briones-Fourzán et al. 2012 [14]; Huchin-Mian et al. 2013 [15]; Davies et al. 2019 [16]) have shown that prevalence of PaV1 is significantly higher in seagrass habitats even after accounting for the significant effect of lobster size, i.e., that irrespective of size, lobsters have a higher probability of infection in seagrass habitats compared to other habitats.**

9. Lines 93-103: these are presumably the objectives, but they are not well stated. They are too vague and loosely constructed to provide a logical flow to the ms. [Lines 472-473 give yet another objective. To tackle the notion of alternate hosts for the virus, one should “find and grind” many animals in the seagrass to see if any are overtly positive. One coudl also do more infection trials. These remain to be done in this system.]

**R. We agree that the objectives were not well stated and constructed. The paragraph containing the objectives has been completely rewritten (lines 81–97 in revised ms). About the comment between brackets: Yes; that is the idea for future studies: running PCR assays in a sample of the suggested species to find out if any individuals are positive for PaV1.**

10. Lines 68-79: delete this paragraph, superfluous.

**R. This paragraph and all references to *C. solearis* have been deleted**

11. Line 86: strike “relatively”. Ecosystem effects are unknown. Line 80: use a hyphen rather than a “/” when discussing host-parasite systems.

**R. Changed as suggested.**

12. Line 143: why was 50 cm selected as the reference distance between points?

**R. Points must be equally spaced along the transect and to have sufficient points per transect so as to be able to estimate percentages of cover. For comparative purposes, we used the same distance between points as Davies et al. (2019) [16].**

13. Lines 216-248: which variables are dependent variables and which are independent variables in the multitude of statistical analyses? Spell it out.

**R. The main factors are the independent variables. We have now changed the term “main factors” to “independent variables”.**

14. Lines 290, 293, 342, and elsewhere: Put emphasis on the finding, not on the statistic. Could rewrite as: Zone A had the lowest average HAS value and Zone B had the highest (ANOVA, Tukey’s HSD). “Results of GLMs showed that” this is an empty phrase. We’re interested in the biological finding.

**R. OK. Throughout the Results section, emphasis has now been put in the finding, supported by the statistic. Thanks for the suggestion.**

15. Lines 361-368: delete this section on C. solearis. Data are too sparse. Pick up at line 369.

**R. The work on *C. solearis* has been removed from the manuscript.**

16. Line 404: the 95% confidence intervals: are these based on the quadrat data or the prevalence? If the latter then it’s a binomial (0/1) and the sd = square root (npq), thus the CIs would be smaller.

**R. The 95% confidence intervals are based on prevalence, which is a percentage (i.e., proportion x 100). As explained in lines 246-248 (Materials and Methods): “clinical prevalence (the percentage of clinically infected lobsters) was estimated for each sampling period, and 95% confidence intervals were computed using Wilson’s score method with continuity correction [55]”. = Newcombe (1998).**

17. Discussion shows an important problem in the design on line 410: “seemingly different characteristics were selected”. A pilot study or earlier work in the region might have uncovered this issue. The zones weren’t different because the habitat was 73% seagrass in each zone; i.e., they're not very different.

**R. Actually, our choice of sampling zones was based on the findings of many earlier studies. This has been clarified in the revised ms (new line 101-115). The Puerto Morelos reef lagoon is a UNESCO’s CARICOMP (Caribbean Coastal Marine Productivity Program) site and has been extensively studied for decades (e.g., van Tussenbroek 1995, 1998, 2011; Ruiz-Rentería et al. 1998; Enríquez and Pantoja-Reyes 2005; Rodríguez-Martínez et al. 2010; van Tussenbroek et al. 2014; Zarco-Perelló and Enríquez 2019) [29–36]. These studies have consistently recognized three lagoon zones based on different characteristics of the vegetation: (1) a narrow coastal fringe, (2) a broad mid-lagoon zone, and (3) an area of back-reef vegetation. In the broad mid-lagoon zone, differences have been found in some areas, such as those where our zones B and C were located (Enríquez and Pantoja Reyes 2005 [33]; van Tussenbroek 2011 [31]). Our zone A was located in the area of back-reef vegetation. Although our zones did not differ in the invertebrate community composition, they did differ in habitat complexity and % cover of most substrates (see Table 1), and some of these characteristics also varied with sampling period. However, unlike in Davies et al. (2019) [16], where differences between a reef lagoon and a back-reef site in Bahía de la Ascensión were larger and related with PaV1 prevalence, the differences in habitat characteristics in our sampling zones at Puerto Morelos did not appear to be sufficiently large so as to influence prevalence of PaV1.**

18. Was temporal variation important? Was spatial variation important? Were there differences in this seemingly homogeneous habitat? These were investigated but they weren't presented well. A bigger question might be were their differences in lobster density within the zones and did this affect prevalence?

**R: Both spatial and temporal variation was important for habitat complexity (HAS values) and the abundance (% cover) of substrates such as seagrass, macroalgae, rubble, and sponges, but not for the composition of the invertebrate communities or for PaV1 prevalence. Unfortunately, we cannot provide estimates of lobster density because we do not have an accurate estimate of the area of the zones that were surveilled for lobsters.**

19. Lines 436-438: See above points regarding seagrass as an “environmental reservoir”. Also important would be host effects. I can think of many that could contribute to the host surviving longer in areas with more cover, easier access to food, etc. Host factors are important here and the predilection for the smaller animals may not be the only one.

**R: On the comment about seagrass as an “environmental reservoir”, please refer to response to comment 7. On the rest of the comment, we are not sure what the reviewer is referring to, as these lines make no reference to host survival.**

20. Lines 510: not sure I agree with reference #28, stressors are known to increase pathogens that are host specialists as well as generalists. Vector-borne diseases are good examples. I think the issue is direct vs. indirect life cycles rather than host specificity. Suggest deleting or re-writing.

**R. Reference to former reference #28 has been removed from this paragraph.**

21. Table 2: the indices really should be labeled in the table heading.

**R. OK. Indices have been labeled in the table heading.**

22. Figure 2: not sure that this figure contributes much to the Results or Discussion, particularly given Figure 3. Suggest using one or the other, but not both. I’d go with Figure 3 and I’d probably use stacked bar graphs with the means data, but that may not be appropriate.

**R. We respectfully disagree. Figs 2 and 3 express different results. It is recommended combining multivariate analyses (considered more as exploratory analyses) and univariate analyses (which are tests of hypotheses). Fig 2 provides information on the relative importance of different types of substrates driving differences among zones over time, whereas Fig 3 shows the mean ± 95% CI for each substrate per zone over time.**

23. Figure 4: this might be best as supplemental data. There is very little variation in this data and I wonder if the statistical analysis had enough power to give any credence to the significant differences shown. This should be discussed.

**R. We have moved Fig 4 to supporting information. However, we are not sure about the reference to power. Fig 4 (now S1 Fig) shows the mean ± 95% CI of HAS, by zone and period. In the GLM, the effect of both factors (i.e., independent variables) was significant; therefore, the null hypothesis (of no difference among means) was rejected. Power analysis is recommended when the null hypothesis is not rejected, but even in those cases some statisticians (e.g., Hoenig & Heisey 2001) argue against its use and in favor of putting more emphasis on the investigator's choice of hypotheses and on the interpretation of confidence intervals.**

24. Figure 5: I would use either Figure 4 or Figure 5 (I prefer Fig 5) to show that there is little separation in the habitats. Could do this for infected vs. uninfected animals and see if there are differences?

**R. Fig 4 (mean ± 95% CI of HAS, by zone and period) has been moved to supporting information (new S1 Fig). However, Fig 5 depicts the results of multivariate analyses on invertebrate community composition, so we are not sure what the reviewer means by “could do this for infected vs uninfected animals and see if there are differences?” Unless the reviewer means something similar to former Fig 4, in which case please see response to comment 26 below.**

25. Figure 6: the clinical prevalence is lower than might be expected here, only hitting 4% at 30 mm CL. By the way, is a 73 mm CL lobster still a juvenile?

**R. The overall average clinical prevalence was 16.2%. In Fig 6 (now Fig 5), the entire orange section represents all clinically infected lobsters. The figure shows a mode in the 30 mm CL size class, with 18.3% of the total lobster sample, of which about 1/5 were diseased (i.e., 3.8% of the entire lobster sample). Along the Mexican Caribbean *P. argus* lobsters mature at about 75 to 80 mm CL (see Fonseca-Larios and Briones-Fourzán 1998), so a 73 mm CL lobster is still sexually immature (i.e., juvenile).**

26. I would add an additional figure here. Shouldn’t there be a figure showing prevalence of PaV1 by site*time. (I was expecting something akin to Figure 7 but with prevalence data). I know this has been done in other studies of PaV1 from the region.

**R. The first logistic model showed that probability of clinical infection was not affected by zone; i.e., the significant differences in habitat complexity and in % cover of substrates among zones were not related with the prevalence of PaV1. Therefore, it was possible to pool data of lobsters from all zones to have a global, local estimation of prevalence. Regardless, an additional figure of prevalence akin to former Fig 7 is now in the supporting information (S4 Fig), where former Fig 4 (now S1 Fig) and Fig 7 (now S3 Fig) were moved, as suggested by the reviewer.**

27. Figure 7 could be deleted with no loss to the main points.

**R. OK. Fig 7 has been moved to supporting information (now S3 Fig).**

28. Figure 8: see comments regarding the estimation of standard deviations from binomial data. I presume these are mean values from transects or zones, grouped and analyzed collectively. If that’s the case they are not binomial data, and the sample size isn’t reflective of the zone or transect number.

**R. The 95% confidence intervals for prevalence were not estimated from binomial data (i.e. 1/0); they were estimated from binomial proportional data (percentages). See Newcombe (1998) [55] or Agresti & Coull (1998). As mentioned in the previous response, results of the logistic regression models made it possible to pool data of lobsters from all zones to have a global, local estimation of prevalence. Pooling the lobsters from all zones has the advantage of increasing sample size, which decreases the width of the 95% CI, thus providing a more accurate estimation of prevalence for monitoring purposes over the longer term.**

Minor style points:

29. Line 28: “and/or”. Just use one or the other, not both. “Or” usually works best.

**R: Sentence had been rewritten.**

30. Line 51: “structured crevice-type shelters”. This is jargon. It’s structured habitats or crevice-like shelters or dens.

**R: OK, “structured” has been removed.**

31. Line 149: Re the habitat complexity estimates: these are probably more subjective than qualitative.

**R: The estimates were made by scientists who have been doing them for several years, so though qualitative, they are not subjective.**

32. Line 162: give minimum size of animals identified in the biodiversity component. Was it >10 cm or >10 mm or some other value?

**R: The minimum size has been added (>1 cm).**

33. Line 199: American spelling was used throughout except for “Haemolymph”. Consistency.

**R: OK. Thanks for catching this.**

34. Line 216: “percent data on the cover” is jargon. “data on the percent cover” is not. For statistical purposes, in logit-transformed data, I presume it’s presence/absence (0/1) data that are being analysed as in a logistic regression?

**R: Sentence has been changed to “data on percent cover”. In this case, the data being analyzed is the percentage of cover (not the presence/absence) of the different substrates. Percentages (or proportions) are not normally distributed and need to be properly transformed to be analyzed. The arcsine square root transformation is the most common transformation of proportions, but Warton & Hui (2011) [48] argue that it is much better to transform proportions to logits. The logit transform of a proportion is: log (p/[1–p]) (where p = proportion). However, because the logit transform for proportions equal to 0 and 1 are the undefined values -∞ and ∞, respectively, an ad hoc solution is to add some small value ε to both the numerator and denominator of the logit function. Warton & Hui [48] propose taking as ε the minimum non-zero proportion (or if proportions are large, the minimum non-zero value for 1–p).**

35. Line 233: why was square-root transformation used? I know why, but mention briefly to readers, i.e., to adjust variance to meet assumptions of normality.

**R: Transformation serves a different purpose in multivariate analyses for communities than in univariate analyses. In biological communities, some species may be very abundant and some may be rare. This affects the computation of the similarity (Bray-Curtis) index matrix, but may be alleviated by transforming the data. In ordination analyses (e.g. nMDS), the data transform sequence is: no transform, square root, fourth root (or log (x+1)), and presence/absence. These transformations shift the focus from the dominant species (no transform) to the rarer species (presence/absence), so when one has quantitative data (and not only presence/absence data), the root or fourth root transforms are recommended. These transforms have the effect of down-weighting the importance of the highly abundant species, so that similarities depend not only on their values but also on less common species. In other words, the root transform retains the quantitative information while downplaying the species dominants (Clarke & Warwick 2001) [53]. This last sentence has been added in the revised ms (new lines 233-234).**

36. Line 494-495: rewrite this sentence. There are well known means to adjust clinical prevalence levels using sensitivity and specificity.

**R. Yes there are, but as shown by Pestal et al. (2003), in the special case where specificity equals 1.0, then the estimation of the proportion of “truly infected” organisms is reduced to the estimated prevalence based on macroscopic criteria over the sensitivity estimate. In this case, the sensitivity of clinical signs was 0.5 (i.e., for each lobster with clinical signs there is another one infected but with no clinical signs) and specificity was 1.0 (i.e., all lobsters with clinical signs were positive for PaV1 by PCR. Therefore, multiplying clinical prevalence by 2 can provide a rough estimation of real prevalence (Huchin-Mian et al. 2013 [15]; Candia-Zulbarán et al. 2019 [46]).**

37. Line 546: should spell out this journal reference.

**R. This and other references on *Cymatocarpus solearis* have been removed.**

**Reviewer #2: Summary:**

This manuscript describes an ambitious study aimed at determining whether there are habitat or community characteristics that can explain the prevalence of the virus PaV1 or the digenean trematode Cymatocarpus solearis among Caribbean spiny lobsters in a tropical reef lagoon offshore of Puerto Morelos, Mexico. The authors did not find any consistent associations between any of the characteristics they measured and the prevalence of these pathogens, other than one spike in PaV1 prevalence during one of the sampling periods.

General comments:

Overall the manuscript was well written and well organized. The abstract needs revision and the introduction needs some reorganization and revision (marked copy of these sections is attached), but the remainder of the manuscript text was clear and easily interpreted. The approach seemed appropriate, as were the statistical analyses and interpretation. The main problem with the manuscript is the lack of any consequential, significant findings. The only significant finding was a bump up in prevalence of PaV1 during one sampling period and this does not create any kind of pattern for interpretation (and the authors recognized this). This is always a difficult position to be in and I applaud the authors for writing the paper regardless because the lack of patterns or associations is still important to publish because, if for nothing else, it keeps other researchers for attempting similar studies that are apt to find the same results.

1. The sampling zones seem rather close to one another. I wonder if the proximity of the zones to one another doesn’t allow the lobsters to easily move between them and effectively ameliorate any effect of habitat or community characteristics on prevalence of these pathogens? I suggest that this be addressed in the discussion.

**R. Thanks for calling our attention to this potential misinterpretation of the data. The sampling zones were chosen based on different attributes of the reef lagoon benthic habitats (see response 17 to Reviewer 1). The population of *P. argus* in the reef lagoon comprises mainly juveniles and the distance between zones ranged between 600 m and 1 km. These distances exceed the movement ranges of juvenile *P. argus* ≤50 mm CL as assessed in sites enhanced with casitas in this same reef lagoon (<100 m) (Briones-Fourzán et al. 2007) [27]. This information has been added in the revised manuscript.**

Specific comments:

2. PDF is attached with suggested edits to title, abstract, and introduction. Remainder of the manuscript was much better.

**R. Suggestions in the PDF attached by Reviewer 2 have been taken into account. The title has been changed, and the abstract and introduction have undergone substantial rewriting. All mention to the work on *C. solearis* was removed from the manuscript.**

3. Note: ignore the bracketed comment on lines 68-79. At that point I thought the research was focused solely on PaV1 since C. solearis is not mentioned in the abstract at all (fix that)!

**R. The work on *C. solearis* was removed from the manuscript to focus on the PaV1 results.**

4. Line 172 Give a rationale for including the additional lobster sampling periods

**R. OK. Lobster samplings were continued in order to monitor the prevalence of the disease in this reef lagoon system over the long term. This has now been clarified in the revised manuscript.**

5. It’s confusing the way the tables are nested in the manuscript with the captions following them, and the figure captions within the manuscript text. Are they not supposed to be after the literature cited?

**R. Nesting the tables in the manuscript with the captions following them, and putting the figure captions where the figures are suggested to be placed, are requested by PLoS ONE in the Instructions to Authors.**

6. Figures and table are nice and clearly constructed.

**R. Thank you!**

**WE THANK REVIEWERS 1 AND 2, AND THE ACADEMIC EDITOR FOR THEIR THOROUGH REVIEWS AND VALUABLE COMMENTS**

6. PLOS authors have the option to publish the peer review history of their article ([what does this mean?](https://journals.plos.org/plosone/s/editorial-and-peer-review-process#loc-peer-review-history)). If published, this will include your full peer review and any attached files.

If you choose “no”, your identity will remain anonymous but your review may still be made public.

**Do you want your identity to be public for this peer review?** For information about this choice, including consent withdrawal, please see our [Privacy Policy](https://www.plos.org/privacy-policy).

Reviewer #1: No

Reviewer #2: No

**References to papers cited in our Responses that are not in the revised manuscript:**

Agresti A, Coull BA. 1998. Approximate is better than "Exact" for interval estimation of binomial proportions. Am Stat 52: 119–126.

Fonseca-Larios, Briones-Fourzán P. 1998. Fecundity of the spiny lobster *Panulirus argus* (Latreille, 1804) in the Caribbean coast of Mexico. Bull Mar Sci 63: 21–32.

Hoenig JM, Heisey DM. 2001. The abuse of power: the pervasive fallacy of power calculations for data analysis. Am Stat 55: 1–6.

Pestal GP, Taylor DM, Hoenig JM, Shields JD, Pickavance R. 2003. Monitoring the prevalence of the parasitic dinoflagellate *Hematodinium* sp. in snow crabs *Chionoecetes opilio* from Conception Bay, Newfoundland. Dis Aquat Org 53: 67−75.

Sweet MJ, Bythell JC, Nugues MM. 2013. Algae as reservoirs for coral pathogens. PLoS ONE 8(7): e69717.

Raymond B, Hartley SE, Cory JS, Hails RS. 2005. The role of food plant and pathogen-induced behaviour in the persistence of a nucleopolyhedrovirus. J Invertebr Pathol 88: 49–57.

[NOTE: If reviewer comments were submitted as an attachment file, they will be attached to this email and accessible via the submission site. Please log into your account, locate the manuscript record, and check for the action link "View Attachments". If this link does not appear, there are no attachment files to be viewed.]

While revising your submission, please upload your figure files to the Preflight Analysis and Conversion Engine (PACE) digital diagnostic tool, <https://pacev2.apexcovantage.com/>. PACE helps ensure that figures meet PLOS requirements. To use PACE, you must first register as a user. Registration is free. Then, login and navigate to the UPLOAD tab, where you will find detailed instructions on how to use the tool. If you encounter any issues or have any questions when using PACE, please email us at [figures@plos.org](https://correo.cmarl.unam.mx/correo/src/compose.php?send_to=figures%40plos.org). Please note that Supporting Information files do not need this step.
