# Supplementary material for: Do ecological characteristics drive the prevalence of Panulirus argus virus 1 (PaV1) in juvenile Caribbean spiny lobsters in a tropical reef lagoon?
Source: PLoS One. 2020 Feb 28;15(2):e0229827. doi: 10.1371/journal.pone.0229827 (PMC7048287; doi:10.1371/journal.pone.0229827)
Supplement: Attachment — Submitted filename: PLoS editorial comment_Juvenile lobster disease R1.docx [file pone.0229827.s009.docx]

**PLoS editor comments_Juvenile lobster disease prevalence R1**

Lines 23-26: A bit of a run on sentence and confusing as written. I would break it up with a full stop after ‘2001’ on line 25 and start the next sentence ‘Disease prevalence has been irregularly assessed since 2001, but levels have increased between 2001 and 2006, and then varied between 2009 and 2014.’

Line 35: I would offset the i.e. text with parentheses.

Line 46: I don’t think ‘wider’ should be capitalised

Line 64: Should be ‘highly-vegetated’

Line 68: What parasite? This is confusing as PaV1 is a virus so this sounds like something else is being discussed. Keep the terminology consistent.

Line 79: Delete ‘also’

Line 83: Write as ‘for juvenile P. argus’

Line 88: Should be ‘small-scale habitats’

Line 110: Should be ‘greatest part’ or ‘majority’

Line 114: Write as ‘there is more hard substrate’

Line 117: Again, write as ‘for small juvenile P. argus’; the ‘of’ is not necessary

Line 122: Write as ‘and later on PaV1 disease dynamics’

Line 223: Do not capitalise ‘general linear models’

Line 243: The abbreviation ANOSIM only seems to appear one more time. The PLoS rule is that abbreviations must be used at least 3 times, otherwise they should not be introduced so this abbreviation should be deleted.

Line 269: Use the abbreviation GLM

Line 316: Spell out ANOSIM

Line 327: Use the abbreviation GLM

Line 334: Use only the abbreviation nMDS which has already been defined and does not need to be defined here again.

Line 356: The sub-heading should be on line 357

Line 403: Should be ‘in the Puerto Morelos reef lagoon’

Lines 403-408: This is all just repeating the methods, which is not necessary

Line 412: I don’t think the i.e.’ makes sense here and the text that follows is too long.. I would put a full stop after’ infection’ and start the next sentence ‘Although there were….’

Line442: Write as ‘as juvenile P. argus’
