# Supplementary material for: Do ecological characteristics drive the prevalence of Panulirus argus virus 1 (PaV1) in juvenile Caribbean spiny lobsters in a tropical reef lagoon?
Source: PLoS One. 2020 Feb 28;15(2):e0229827. doi: 10.1371/journal.pone.0229827 (PMC7048287; doi:10.1371/journal.pone.0229827)
Supplement: Attachment — Submitted filename: Response to Review comments.docx [file pone.0229827.s010.docx]

**Date:**	Feb 03 2020 08:22AM

**To:**	"Patricia Briones-Fourzán" [briones@cmarl.unam.mx](mailto:briones@cmarl.unam.mx)

**From:**	"PLOS ONE" [plosone@plos.org](mailto:plosone@plos.org)

**Subject:**	PLOS ONE Decision: Revision required [PONE-D-19-25184R1]

**Attachment(s):** [PLoS editorial comment_Juvenile lobster disease R1.docx](https://www.editorialmanager.com/pone/download.aspx?guid=%7Bb13cac90-5e80-4471-987e-614430e09c61%7D&amp;scheme=13&amp;id=396856)

PONE-D-19-25184R1

Ecological determinants in the prevalence of Panulirus argus virus 1 (PaV1) in juvenile Caribbean spiny lobsters in a tropical reef lagoon

PLOS ONE

Dear Dr. Briones-Fourzán,

Thank you for submitting your manuscript to PLOS ONE. After careful consideration, we feel that it has merit but does not fully meet PLOS ONE’s publication criteria as it currently stands. Therefore, we invite you to submit a revised version of the manuscript that addresses the points raised during the review process.

I think the authors have done a good job addressing most of the comments of the reviewers and the reviewer generally agreed (note that the second reviewer was not available to assess the revision). The reviewer and I have both provided some minor comments. However, I agree with the reviewer in that it is possible, if not likely, that the design of the study contributed to the results and a future study should use sites that are much further apart. Both reviewers made this comment during the first round of review, and I agree, so the authors need to address it in some way in the manuscript. I think at the very least the authors need to acknowledge this as a potential issue with the current study and advice for a future study.

**R: The potential issue has been acknowledged. Please see response to comments by Reviewer # 2 below.**

We would appreciate receiving your revised manuscript by Mar 19 2020 11:59PM. When you are ready to submit your revision, log on to https://www[.editorialmanager.com/pone/](http://www.editorialmanager.com/pone/) and select the 'Submissions Needing Revision' folder to locate your manuscript file.

If you would like to make changes to your financial disclosure, please include your updated statement in your cover letter. To enhance the reproducibility of your results, we recommend that if applicable you deposit your laboratory protocols in protocols.io, where a protocol can be assigned its own identifier (DOI) such that it can be cited independently in the future.

For instructions see: <http://journals.plos.org/plosone/s/submission-guidelines#loc-laboratory-protocols>

Please include the following items when submitting your revised manuscript:

A rebuttal letter that responds to each point raised by the academic editor and reviewer(s). This letter should be uploaded as separate file and labeled 'Response to Reviewers'.

A marked-up copy of your manuscript that highlights changes made to the original version. This file should be uploaded as separate file and labeled 'Revised Manuscript with Track Changes'.

An unmarked version of your revised paper without tracked changes. This file should be uploaded as separate file and labeled 'Manuscript'.

Please note while forming your response, if your article is accepted, you may have the opportunity to make the peer review history publicly available. The record will include editor decision letters (with reviews) and your responses to reviewer comments. If eligible, we will contact you to opt in or out.

We look forward to receiving your revised manuscript. Kind regards,

Heather M. Patterson, Ph.D. Academic Editor

PLOS ONE

[Note: HTML markup is below. Please do not edit.] Reviewers' comments:

Reviewer's Responses to Questions

**Comments to the Author**

1. If the authors have adequately addressed your comments raised in a previous round of review and you feel that this manuscript is now acceptable for publication, you may indicate that here to bypass the “Comments to the Author” section,

enter your conflict of interest statement in the “Confidential to Editor” section, and submit your "Accept" recommendation. Reviewer #2: (No Response)

2. Is the manuscript technically sound, and do the data support the conclusions?

The manuscript must describe a technically sound piece of scientific research with data that supports the conclusions. Experiments must have been conducted rigorously, with appropriate controls, replication, and sample sizes. The conclusions must be drawn appropriately based on the data presented.

Reviewer #2: Partly

3. Has the statistical analysis been performed appropriately and rigorously? Reviewer #2: Yes

4. Have the authors made all data underlying the findings in their manuscript fully available?

The [PLOS Data policy](http://www.plosone.org/static/policies.action#sharing) requires authors to make all data underlying the findings described in their manuscript fully available without restriction, with rare exception (please refer to the Data Availability Statement in the manuscript PDF file). The data should be provided as part of the manuscript or its supporting information, or deposited to a public repository. For example, in addition to summary statistics, the data points behind means, medians and variance measures should be available. If there

are restrictions on publicly sharing data—e.g. participant privacy or use of data from a third party—those must be specified. Reviewer #2: Yes

5. Is the manuscript presented in an intelligible fashion and written in standard English?

PLOS ONE does not copyedit accepted manuscripts, so the language in submitted articles must be clear, correct, and unambiguous. Any typographical or grammatical errors should be corrected at revision, so please note any specific errors here.

Reviewer #2: Yes

6. Review Comments to the Author

Please use the space provided to explain your answers to the questions above. You may also include additional comments for the author, including concerns about dual publication, research ethics, or publication ethics. (Please upload your review as an

attachment if it exceeds 20,000 characters)

Reviewer #2: The authors addressed most of my concerns noted in the first review; however, the core problems still exist. The zones that the authors used in the study were rather homogeneous to start and too close together so the lack of a difference in PaV1 was really not surprising. I think the authors did a decent job recognizing the former in the discussion, so I am OK with that. Their other responses to my comments and suggestions are also acceptable.

**R: Thank you for the positive feedback.**

However, in their response to my inquiry about lobster movement between zones, the authors reference a study that shows small juvenile lobsters have a foraging range of < 50m as justification for the proximity of the zones making them independent. As I understand it from that paper, that foraging range is an estimate for a single night (straight line distance from the shelter used one night to the one used on the next night). Thus, over the course of weeks a juvenile lobster could easily shelters as steppingstones to move between the habitat zones in the study, and therefore, move PaV1 between zones. The authors need to mention more explicitly that the proximity between the zones (A & B appear < 500m apart) may have allowed lobsters to mingle and ameliorate the effect of any difference between zones, or something to that effect.

**R: [Just to clarify: In the referenced study (Briones-Fourzán et al. 2007), the foraging range was not an estimate for a single night. It was an estimate based on subsequent recaptures of marked lobsters, which occurred during surveys conducted at two to three-month intervals. Due to the scarcity of natural shelters in the reef lagoon, the average persistence of juveniles <50 mm CL was twice as long in sites with casitas (up to 3 months) as in sites with no casitas (up to 1.5 months).]**

**In the Discussion of the previous ms, after comparing our results with those obtained in Bahía de la Ascensión, we wrote “Therefore, it would appear that the scale of habitat differences required to be associated with a change in disease prevalence requires a larger range of lobster sizes or a wider variation in habitats, such as those studied in Bahía de la Ascensión.” But distance was not explicitly mentioned. Therefore, we have now added the following paragraph (lines 414-421 of revised ms): “Because natural crevice-type shelters for lobsters are very scarce in the Puerto Morelos reef lagoon [39], the sampling zones included experimental sites where casitas were deployed years ago for other studies [25,27,39,40]. Casitas increase density of juvenile lobsters as well as their persistence in a site [27], and the distance between our sampling zones was greater than the average movement ranges of juvenile *P. argus* [2,26,27]. Yet, it cannot be dismissed that some mingling of lobsters could occur over time, potentially masking any effect of habitat characteristics on PaV1 prevalence. Therefore, future studies should use sampling sites that are further apart and, whenever possible, located over more heterogeneous habitats.” This involved shortening the third paragraph (between lines 449 and 451) after the new one to avoid repetition.**

Specific comments:

The title seems to suggest that ecological determinants were found when they really were not. I suggest the title be changed to a question. “Do ecological characteristics drive the prevalence of…?” I say this because I think the problems with the design (proximity between zones and their ecological similarity) might really be why the ecological characteristics that were assessed where not significant drivers of PaV1, not that they might not be significant in another system/design.

**R: Title has been changed as suggested. We also changed the running title.**

The detailed info on the monitoring of PaV1 in the lagoon should be omitted from the abstract. Keep that to one general line.

**R: The abstract has been modified as suggested.**

7. PLOS authors have the option to publish the peer review history of their article ([what does this mean?](https://journals.plos.org/plosone/s/editorial-and-peer-review-process#loc-peer-review-history)). If published, this will include your full peer review and any attached files.

If you choose “no”, your identity will remain anonymous but your review may still be made public.

**Do you want your identity to be public for this peer review?** For information about this choice, including consent withdrawal, please see our [Privacy Policy](https://www.plos.org/privacy-policy).

Reviewer #2: No

[NOTE: If reviewer comments were submitted as an attachment file, they will be attached to this email and accessible via the submission site. Please log into your account, locate the manuscript record, and check for the action link "View Attachments". If this link does not appear, there are no attachment files to be viewed.]

While revising your submission, please upload your figure files to the Preflight Analysis and Conversion Engine (PACE) digital diagnostic tool, <https://pacev2.apexcovantage.com/>. PACE helps ensure that figures meet PLOS requirements. To use PACE, you must first register as a user. Registration is free. Then, login and navigate to the UPLOAD tab, where you will find detailed instructions on how to use the tool. If you encounter any issues or have any questions when using PACE, please email us at [figures@plos.org](mailto:figures@plos.org). Please note that Supporting Information files do not need this step.

[*In compliance with data protection regulations, you may request that we remove your personal registration details at any time. (Remove my information/details). Please contact the publication office if you have any questions.*](https://www.editorialmanager.com/pone/login.asp?a=r)
